# Variants in *ATP6V0A1* cause progressive myoclonus epilepsy and developmental and epileptic encephalopathy

**DOI:** 10.1093/braincomms/fcab245

**Published:** 2021-10-18

**Authors:** Laura C Bott, Mitra Forouhan, Maria Lieto, Ambre J Sala, Ruth Ellerington, Janel O Johnson, Alfina A Speciale, Chiara Criscuolo, Alessandro Filla, David Chitayat, Ebba Alkhunaizi, Patrick Shannon, Andrea H Nemeth, Italian Undiagnosed Diseases Network, Domenica Taruscio, Marco Salvatore, Agata Polizzi, Federica Censi, Giovanna Floridia, Giuseppe Novelli, Erica Daina, Alessandra Ferlini, Marcella Neri, Dario Roccatello, Simone Baldovino, Elisa Menegatti, Francesco Angelucci, Wooi Fang Lim, Pasquale Striano, Federico Zara, Ingo Helbig, Mikko Muona, Carolina Courage, Anna-Elina Lehesjoki, Samuel F Berkovic, ATPase Consortium, Nancy Pinnell, Dallas Reed, Peter D Turnpenny, Jacqueline Eason, Leah Fleming, Kirsty McWalter, Kali Juliette, Paul J Benke, Xilma Ortiz-Gonzalez, Sarah Mckeown, Amisha B Patel, Matthew Osmond, Jagdeep S Walia, Xianru Jiao, Zhixian Yang, Boris Keren, Charles Perrine, Ashish Deshwar, Kenneth H Fischbeck, Francesco Brancati, Richard I Morimoto, Matthew J A Wood, Carlo Rinaldi

**Affiliations:** 1 Department of Molecular Biosciences, Rice Institute for Biomedical Research, Northwestern University, Evanston, IL 60208, USA; 2 Department of Paediatrics, University of Oxford, Oxford OX1 3QX, UK; 3 Department of Physiology, Anatomy and Genetics, Oxford OX1 3QX, UK; 4 Department of Neurosciences, Reproductive and Odontostomatological Sciences, Federico II University, Naples 80121, Italy; 5 Neuromuscular Diseases Research Section, Laboratory of Neurogenetics, National Institute on Aging, National Institutes of Health, Bethesda, MD 20892, USA; 6 Division of Clinical and Metabolic Genetics, Department of Pediatrics, The Hospital for Sick Children, University of Toronto, Toronto, Ontario M5G 1X8, Canada; 7 The Prenatal Diagnosis and Medical Genetics Program, Department of Obstetrics and Gynecology, Mount Sinai Hospital, University of Toronto, Toronto, Ontario M5G 1X5, Canada; 8 Department of Pathology and Laboratory Medicine, Mount Sinai Hospital, University of Toronto, Toronto, Ontario M5G 1X5, Canada; 9 Nuffield Department of Clinical Neurosciences, University of Oxford, Oxford OX3 9DU, UK; 10 Department of Life, Health and Environmental Sciences, University of L'Aquila, 67100 Coppito, L'Aquila, Italy; 11 Institute for Research, Hospitalization and Health Care (IRCCS) “G. Gaslini” Institute, Genova 16147, Italy; 12 Division of Neurology, Children’s Hospital of Philadelphia, Philadelphia, PA 19104, USA; 13 The Epilepsy NeuroGenetics Initiative (ENGIN), Children’s Hospital of Philadelphia, Philadelphia, PA 19104, USA; 14 Department of Biomedical and Health Informatics (DBHi), Children’s Hospital of Philadelphia, Philadelphia, PA 19104, USA; 15 Department of Neurology, University of Pennsylvania, Perelman School of Medicine, Philadelphia, PA 19104, USA; 16 Blueprint Genetics, 02150 Espoo, Finland; 17 Folkhälsan Research Center, Helsinki, Finland; 18 Department of Medical and Clinical Genetics, Medicum, University of Helsinki, Helsinki, 00290, Finland; 19 Epilepsy Research Centre, Department of Medicine, University of Melbourne, Austin Health, Heidelberg, Victoria 3010, Australia; 20 Neurogenetics Branch, National Institute of Neurological Disorders and Stroke, National Institutes of Health, MD 20892, USA; 21 IRCCS San Raffaele Pisana, 00163 Roma, Italy; 22 Oxford Harrington Rare Disease Centre, University of Oxford, Oxford OX1 3QX, UK

**Keywords:** V-ATPase, epileptic encephalopathy, *Caenorhabditis elegans* disease modelling, organelle acidification, lysosomal disease

## Abstract

The vacuolar H^+^-ATPase is a large multi-subunit proton pump, composed of an integral membrane V0 domain, involved in proton translocation, and a peripheral V1 domain, catalysing ATP hydrolysis. This complex is widely distributed on the membrane of various subcellular organelles, such as endosomes and lysosomes, and plays a critical role in cellular processes ranging from autophagy to protein trafficking and endocytosis. Variants in *ATP6V0A1*, the brain-enriched isoform in the V0 domain, have been recently associated with developmental delay and epilepsy in four individuals. Here, we identified 17 individuals from 14 unrelated families with both with new and previously characterized variants in this gene, representing the largest cohort to date. Five affected subjects with biallelic variants in this gene presented with a phenotype of early-onset progressive myoclonus epilepsy with ataxia, while 12 individuals carried *de novo* missense variants and showed severe developmental and epileptic encephalopathy. The R740Q mutation, which alone accounts for almost 50% of the mutations identified among our cases, leads to failure of lysosomal hydrolysis by directly impairing acidification of the endolysosomal compartment, causing autophagic dysfunction and severe developmental defect in *Caenorhabditis elegans*. Altogether, our findings further expand the neurological phenotype associated with variants in this gene and provide a direct link with endolysosomal acidification in the pathophysiology of *ATP6V0A1*-related conditions.

## Graphical Abstract

**Graphical Abstract:**
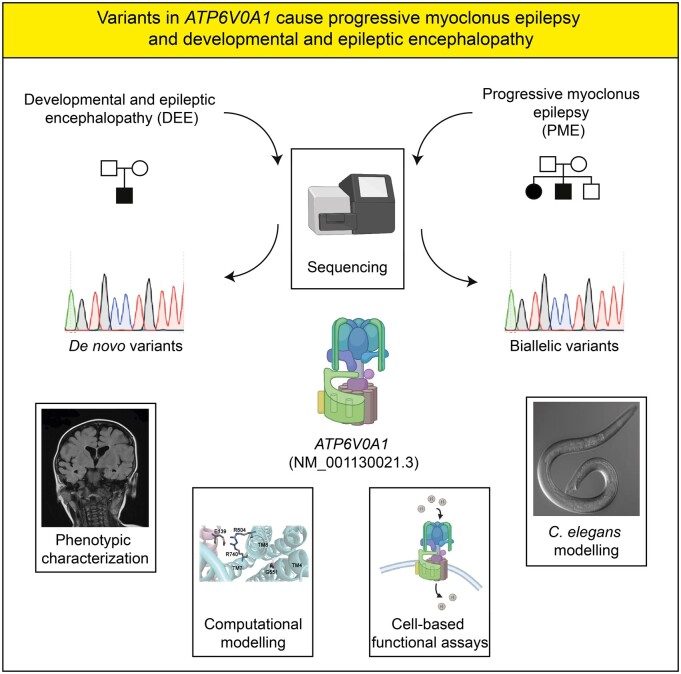

## Teaser

Bott et al. here reports that *de novo* and biallelic variants in *ATP6V0A1* gene affect the ability of the V-ATPase complex to translocate protons and acidify the endolysosomal compartment in neurons, causing a severe neurological phenotype ranging from developmental and epileptic encephalopathy to progressive myoclonus epilepsy.

## Introduction

Vacuolar-type ATPases (V-ATPases) are a ubiquitous, multi-subunit, membrane-embedded rotary proton-pumping motor, arranged into a dissociable peripheral V1 domain (subunits A, B, C, D, E, F, G and H), responsible for ATP hydrolysis, and an integral membrane V0 domain (subunits a, c, c′, c″, d, e), responsible for proton translocation.^1–3^ Through vesicular, luminal and extracellular acidification, the V-ATPases play a critical role in a number of both physiological and pathological cellular processes, from membrane trafficking and substrate degradation in lysosome, to viruses and bacterial toxins internalization and cancer growth and invasion.^4^ Other non-canonical roles of the V-ATPase complex, which are not readily attributable to its proton-moving activity, include membrane fusion, nutrient sensing and scaffold for protein–protein interactions.^5–7^ Recent studies have highlighted the critical importance of the V-ATPase complex in neuronal homeostasis and dysregulation of pH has been shown to be a converging pathogenic mechanism for several diseases, including Alzheimer’s,^8^^,^^9^ Parkinson’s,^10–14^ amyotrophic lateral sclerosis^15^^,^^16^ and lysosomal storage diseases (LSD).^17^^,^^18^ Mutations in subunits of the V-ATPase have been associated with autosomal recessive osteopetrosis (*ATP6V0A3*)^19^^,^^20^ cutis laxa (*ATP6V0A2*),^21^ distal renal tubular acidosis (*ATP6V1B1*, *ATP6V0A4*)^22–24^ and epileptic encephalopathy (*ATP6V1A*),^25^ suggesting that this is an emerging class of human genetic disorders.

Some of the isoforms have different expression patterns in various tissues, which may allow the regulation of V-ATPase in a cell type and subcellular compartment specific manner. *ATP6V0A1* is the brain-enriched isoform of the a subunit in the V0 domain^26^ and is part of the CLEAR (Coordinated Lysosomal Expression and Regulation) network of genes regulated by the master transcription factor EB (TFEB).^27^

Here, we report 5 individuals from 2 unrelated families with compound heterozygous variants in *ATP6V0A1* presenting with a phenotype of early-onset progressive myoclonus epilepsy (PME) and ataxia and 12 cases with *de novo* missense variants in the same gene, with severe developmental and epileptic encephalopathy (DEE), resulting from direct impairment of endolysosome acidification and failure of lysosomal functions.

## Materials and methods

### Patients

Ethical approval was obtained from the ethics committee of the following institutes: University of Federico II (Italy), University of L'Aquila (Italy), G. Gaslini Institute (Italy), University of Exeter (UK), Nottingham University Hospital (UK), Salpêtrière Hospital (France), University of Toronto (Canada), University of Ottawa (Canada), St. Luke's Children's Hospital (USA), Floating Hospital for Children at Tufts Medical Center (USA), Gillette Children's Specialty Healthcare Children's Hospital (USA), Children’s Hospital of Philadelphia (USA), Peking University First Hospital, Beijing (China). Written consent for the study was obtained from patients or legal representatives according to the Declaration of Helsinki. When individuals were not contacted directly, de-identified phenotypic and genomic data were used. Patient or guardian consent was given for the publication of the patient's data and clinical information. Patient II.1 was enrolled in a research study aimed at finding the genetic cause of progressive myoclonus epilepsy; Patient III.1, VII.1-X.1 and XIV.1 were referred to GeneDx for clinical whole-exome sequencing for diagnosis of suspected Mendelian disorders as previously described^28^; Patient IV.1 was recruited in the Italian Undiagnosed Rare Diseases Network (IURDN; PGR00229, 2016–19); Patient V.1 and VI.1 were part of the Deciphering Developmental Disorders Study (DDD; 10/H0305/83). Patient XI.1 was recruited as part of the Care4Rare Canada research study.

### Sequencing and genotyping

**Figure 1:**
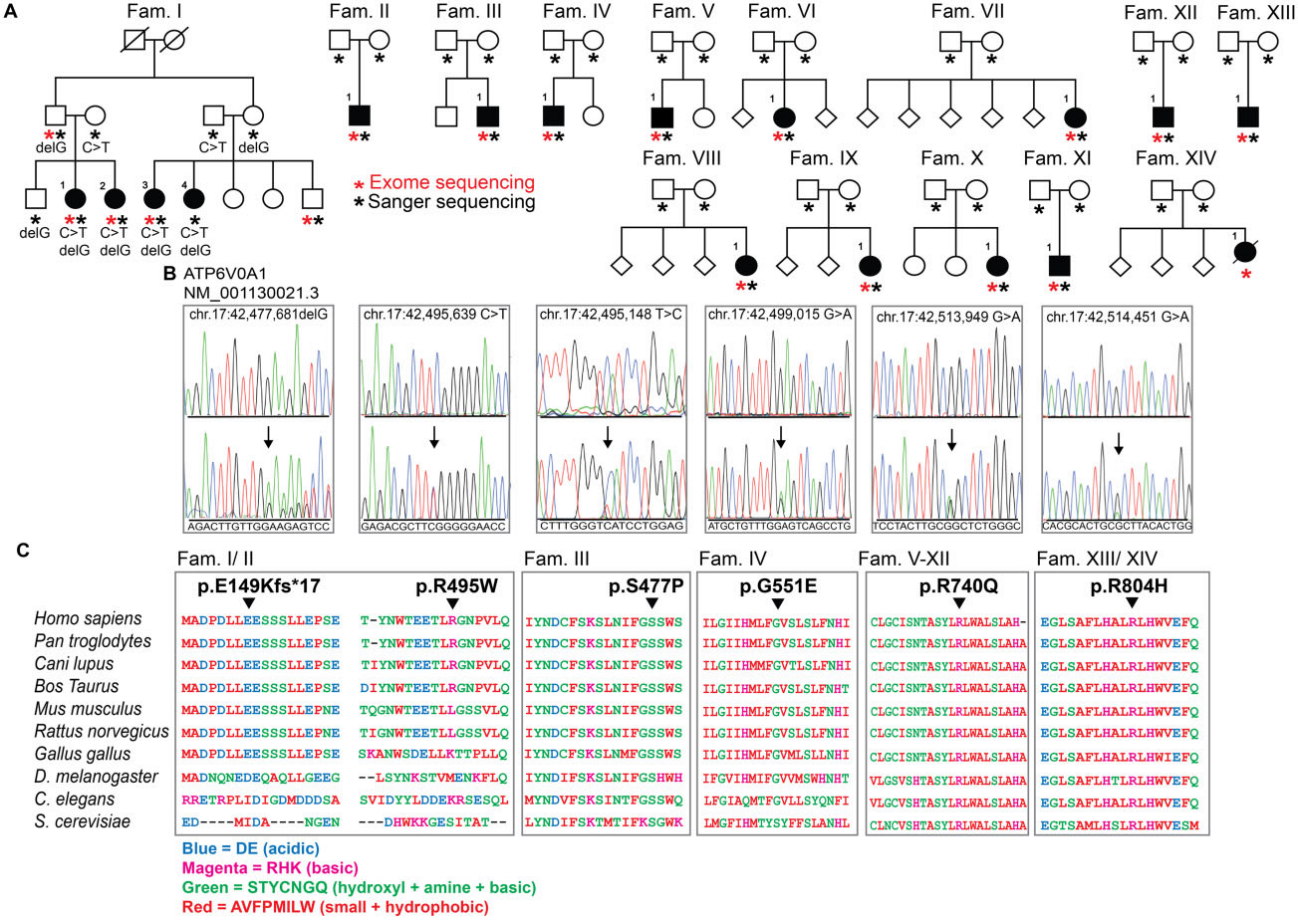
**Identification of disease mutations in *ATP6V0A1*.** (**A**) Pedigrees of all families included in the study are shown. Filled symbols represent affected individuals and are numbered within the family. Asterisks indicate subjects who underwent whole-exome sequencing (red) or confirmatory Sanger sequencing (black). Fam. = family. (**B**) Representative electropherograms of genomic DNA sequencing of unaffected (top panel) and affected (bottom panel) individuals with identified mutations indicated by arrows. Gene symbol, reference sequence and genomic position of the changes are displayed above the electropherograms. (**C**) Sequence alignment of ATP6V0A1 protein across multiple species shows evolutionary conservation of the identified mutated residues, indicated by the black arrowhead, and surrounding regions. Gaps (black lines) are inserted between residues so that identical or similar amino acids are aligned in successive columns. Acidic residues (Asp, Glu) are in blue, basic residues (Arg, His, Lys) are in magenta, uncharged polar amino acids (Ser, Thr, Tyr, Asn, Gln) and Gly and Cys are in green, and nonpolar amino acids aside from Gly and Cys (Ala, Val, Phe, Pro, Met, Ile, Leu, Trp) are in red.

Blood samples were collected and DNA was extracted using standard methods from peripheral blood lymphocytes from the indicated individuals in Fig. 1A.

Fam. I: Exome sequencing was performed as previously described.^29^ Libraries were prepared using the SeqCap EZ Human Exome Library version 2.0 (Roche Nimblegen Inc.) and a 100-base pair paired-end run was performed on the HiSeq 2000, with each sample on a single lane of a TruSeq version 2 flow cell (Illumina). Sequence alignment, quality control and variant calling were performed with BWA, SAMTools, the Genomic Analysis Toolkit and Picard (http://broadinstitute.github.io/picard/ Accessed 21 October 2021). Data analysis was based on the autosomal recessive mode of inheritance. Variant calling and quality score recalibration were performed using GATK (http://www.broadinstitute.org/gatk/ Accessed 21 October 2021). Variants remaining after exome data analysis containing missing data were Sanger sequenced using the BigDye Terminator version 3.1 chemistry (Applied Biosystems), run on an ABI 3730xl analyzer and analysed using Sequencher software version 4.2 (Gene Codes Corporation). Fam. II-XIV: Details on sequencing, alignment, variant calling (inherited and *de novo*) and variant annotation have been described previously.^30–36^ Presence or absence of the disease-causing variants was confirmed on DNA of the proband and additional members from each family by Sanger sequencing.

### Homology modelling and structure analysis of the human V-ATPase

Homology models of the subunit a (NM_001130021.3, NP_001123493.1) and c (NM_001694.4, NP_001685.1) belonging to the V0 domain of the human V-ATPase has been built by Robetta server,^37^ using as templates the 3D structures of the homologous subunits in *Bos Taurus*^38^ (PDB ID: 6XBW; 97% and 98% identity between the homologous subunits a and c). The 3D models of the G551E and S477P mutants have been built with Robetta.^37^ Structural analysis has been carried out with Coot and Pymol (The PyMOL Molecular Graphics System, Version 1.2r3pre, Schrödinger, LLC).

### cDNA cloning and generation of stable cell lines

Human *ATP6V0A1* cDNA was kindly provided by Michael Forgac. Point mutations corresponding to the gene variants identified in the present study were introduced using a QuikChange II site-directed mutagenesis kit (Agilent Technologies) according to the manufacturer’s instructions, and subsequently the constructs were cloned into the PiggyBac (PB) Transposon vector clone (Stratech, PB511B-1). For stable transfection, PB Transposon vector and PB Transposase vector (Stratech, PB210PA-1) were transfected at a ratio of 9:1 into Neuro2a cells plated on 24-well plates (∼50 000 cells per well). One day after transfection, the cells were trypsinized and transferred as serial dilution to fresh tissue culture plates. Drug selection using 10 µg/ml puromycin started on day 3 and was continued for 2–3 weeks until the visible colonies appeared. In order to induce the transgene expression, 1 µg/ml doxycycline was added to the media starting 1 day before transfection.

### Immunofluorescence

After fixation with 4% paraformaldehyde, slides were placed in blocking solution [10% normal goat serum and 0.3% Triton X-100 in phosphate-buffered saline (PBS)] for 45 min at room temperature. Primary antibody staining was done at 4°C overnight in PBS with 5% normal goat serum and 0.1% Triton X-100 (The Dow Chemical Company), using LysoTracker fluorescent dye (L12492, ThermoFischer Scientific) and Cathepsin D antibody (2284, Cell signalling). The slides were then washed 3 times with PBS (0.1% Triton X-100 in PBS), incubated with secondary antibody (Invitrogen, 1:500) for 1 h at room temperature in the dark, and then washed 3 times before drying and adding Vectashield/4′,6-diamidino-2-phenylinodole stain (Vector Laboratories). Coverslips were mounted with Permount (Fisher Scientific). The cells were imaged and acquired using the PANNORAMIC 250 fluorescent microscope scanner (3DHISTECH). An average of 100 cells from 3 independent experiments per each condition were analysed by a blinded investigator.

### Endolysosome pH measurement

Quantification of lysosomal pH was determined using Dextran conjugated Lysosensor Yellow/Blue DND-160 (L7545, Invitrogen). Wild-Type and R740 mutant Neruo2a cells were grown in serum starved conditions (DMEM with antibiotics) with 1 µg/ml doxycycline to ∼80% confluence. Cells were then trypsinized, harvested and aliquoted at 100 µl into a black 96-well microplate with 5 µM Lysosensor probe for 15 min at 37°C with 5% CO_2_ (50 000 cells). The cells were then washed 3× in DMEM and the samples were read in a CLARIOstar Plus spectrophotometer (BMG Labtech) with dual-excitation and dual-emission spectral peaks at 324/441 and 381/541 nm. The ratio of emission was then calculated for each sample as indicated.

### Western blotting and densitometry analysis

Whole cell lysates were collected as previously described.^39^ Protein concentration of whole cell lysates was determined using the Pierce bicinchoninic acid protein assay (23227, Thermo Scientific) with a bovine serum albumin standard curve according to the manufacturer’s protocol. Twenty micrograms of denatured protein were loaded into the precast 4–12% Bis-Tris gels (NP0322BOX, Life Technologies) or 16% Tricine gel (EC6695A, Thermo Scientific) for LC3 immunoblot. The gel was electroblotted onto 0.45 μm nitrocellulose membrane or 0.2 μm PVDF membrane for LC3 western blotting, which was then blocked for 1 h at room temperature with 5% skim milk in PBS containing 0.1% (v/v) Tween-20 and 2% (v/v) serum. The membranes were incubated overnight at 4°C in 1/1000 dilution of following primary antibodies; Notch isoforms using Notch isoform sampler kit (3640, Cell Signalling), Cathepsin D antibody (2284, Cell signalling), LC3 A/B (12741, Cell Signalling), GAPDH (sc-47724) and HRP-linked secondary antibodies. Blots were visualized with chemiluminescence reagent (Life Technologies). Densitometric quantification of bands was performed with the ImageJ software and standardized relative to a loading control against a control protein sample on each blot. Ratios were normalized for each individual experiment, with the wild type sample set as 1.

### *Caenorhabditis elegans* strains and maintenance

*Caenorhabditis* *elegans* were maintained on solid nematode growth medium seeded with *E. coli* OP50 at 20°C using standard methods.^40^ Strains used in this study were Bristol N2 and EG9591 [*unc-32(ox683[unc-32::gfp +loxP])* III].^41^

The insertion of *mcherry* at the 5′ end of endogenous *lgg-1*, and the introduction of a missense mutation corresponding to the substitution of the essential arginine by a glutamine in UNC-32::GFP at position 804 (numbered according to the isoform UNC-32A) into the EG9591 strain, were performed using CRISPR/Cas9 genome editing and resulted in strains AM1219 *lgg-1(rm17[lgg-1::mcherry])* II and AM1233 *unc-32(rm20[unc-32(R804Q)::gfp +loxP])* III*/hT2[bli-4(e937) let-?(q782) qIs48]* (I; III).

### CRISPR/Cas9-mediated genome editing

Genome editing was performed as previously described.^42^ Briefly, Cas9 ribonucleoprotein complexes were assembled in vitro from purified Cas9 (New England Biolabs), tracrRNA and gene specific crRNA (Integrated DNA Technologies), and injected together with a linear DNA repair template in the gonad of adult hermaphrodites. CRISPR editing was performed using *dpy-10* to generate *cn64* rollers as phenotypic marker. Successful edits were identified by PCR screening and verified by Sanger sequencing.

For inserting *mcherry* at the endogenous *lgg-1* locus, we targeted the 5′ end of the first exon with the guide sequence 5′-CCTTCGAATCAAAATGAAGT-3′. The repair template was synthesized by PCR to generate a double-stranded DNA fragment consisting of mCherry amplified from pAP582 (Addgene) and homology overhangs for insertion at the ATG start site using primers 5′-TAACCTTCTCTTCACACTAACCTTCGAATCAAAATGGTCTCAAAGGGTGAAGAAGATAAC-3′ and 5′-CTTCTCAAAGTTGTTCTCCTCCTTGTAAGCCCACTTCTTATACAATTCATCCATGCC-3′. The *lgg-1(rm17)* allele was isolated in a dpy-10(+) background and the resulting strain was back-crossed three times to N2.

For generating the R804Q substitution in UNC-32::GFP, we edited the *unc-32(ox683)* allele using the guide sequence 5′-CTGCTTCATACCTTCGTCTT-3′ and a single-stranded oligonucleotide containing the CGT > CAG change, a silent T > G mutation resulting in the PvuII restriction site for genotyping, and homology arms flanking the edit as repair template 5′-TCTTGGATGTGTGTCACATACTGCTTCATACCTTCagCTgTGGGCTCTTTCATTGGCTCATGCTCGTAAGTAAAG-3′. The resulting *unc-32(rm20)* allele was confirmed by sequencing and kept on the *hT2* balancer for strain maintenance.

### RNA interference

RNAi-mediated knock-down of V-ATPase genes or *lgg-1* was performed by feeding animals with *E. coli* strain HT115(DE3) containing the appropriate RNAi vectors obtained from the Ahringer library and using L4440 as the empty vector control.^43^ Bacterial cultures were grown overnight in LB with 100 µg/ml ampicillin and induced with 5 mM IPTG for 3 h at 37°C before plating. Animals were age-synchronized by a 2-h egg-lay on RNAi plates and collected for analysis 72–96 h later. For RNAi clones that cause developmental defects (*vha-4*, *vha-16* and *vha-17*), the animals were initially grown on L4440 and transferred to RNAi plates at L4 stage.

### Fluorescence imaging

Animals were immobilized in 50 mM sodium azide on 3% agarose pads and imaged using a Zeiss LSM800 confocal microscope with 10× or 20× objective and Zen imaging software. For quantification of LGG-1::mCherry fluorescence levels, maximum intensity projections were generated from Z stack images and fluorescence intensity was quantified by tracing either the nerve ring for UNC-32::GFP or whole animals for LGG-1::mCherry using ImageJ software (NIH).

### Gene expression analysis in *C. elegans*

Age-synchronized populations of at least 100 gravid adults were collected and snap frozen in liquid nitrogen. RNA was extracted using TRIzol (Invitrogen) and purified with QIAGEN RNeasy MinElute columns as per manufacturers’ instructions.^44^ mRNA was reverse transcribed using the iScript cDNA Synthesis Kit (BioRad) and real-time quantitative PCR was performed using iQ SYBR Green Supermix (BioRad) in a BioRad CFX384 Real-Time PCR system. Relative expression was determined from cycle threshold values using the standard curve method and the expression of genes of interest was normalized to *cdc-42*. The primers used are listed in Supplementary Table 2.

### Statistical analysis

Statistical analysis was performed with Graph Pad Prism version 8 for Windows (GraphPad Software, San Diego, CA, USA, www.graphpad.com), using the tests indicated in the figure legends. A standard confidence interval of 95% was applied in all analyses. Displayed in the figure are the mean values of all technical replicates for each of the independent experiments (displayed as single data points). Error bars represent the standard error of the mean. *P*-values <0.05 were considered statistically significant.

### Data availability

The raw data that support the findings of this study are available from the corresponding authors, upon request.

## Results

We have previously identified an Italian family with four members affected by early-onset progressive myoclonus epilepsy (PME) with ataxia and mental retardation, of unknown origin.^45^ Seeking to unravel the genetic cause of this condition, we performed whole-exome sequencing (WES) in 3 affected and 2 unaffected individuals (Fam. I; Fig. 1A, Supplementary Fig. 1) and found two previously unknown, compound heterozygous variants, c.445delG (p.E149Kfs18) and c.1483C>T (p.R495W) in *ATP6V0A1* (NM_001130021.3), that co-segregate with the clinical phenotype (Fig. 1A). From an independent WES screening of 85 PME patients, we identified 1 individual also from Italy with overlapping clinical features, harbouring the same compound heterozygous mutations (Fam. II.1; Fig. 1A, Table 1). Identity by descent analysis showed that the two families are unrelated (PI-HAT value = 0). While the c.445delG is not found in the gnomAD database,^46^ the allelic carrier frequency of the c.1483C>T variant (rs781278654) is 0.00001315 in the control population and predicted to be damaging (PolyPhen score: 0.99). Neither variant was present in an independent cohort of 200 Italian subjects, suggesting low frequency in this population.

**Table 1:** Clinical characteristics

€	P:I.1-4	P:II.1	P:III.1	P:IV.1	P:V.1	P:VI.1	P:VII.1	P:VIII.1	P:IX.1	P:X.1	P:XI.1	P:XII.1	P:XIII.1	P:XIV.1	From: Aoto et al. Individual 1	From: Aoto et al. Individual 2	From: Aoto et al. Individual 3	From: Aoto et al. Individual 4
*ATP6V0A1* variants	c.445delG; c.1483C>T	c.445delG; c.1483C>T	c.1429T>C	c.1652G>A	c.2219G>A	c.2219G>A	c.2219G>A	c.2219G>A	c.2219G>A	c.2219G>A	c.2219G>A	c.2219G>A	c.2411G>A	c.2411G>A	c.2219G>A	c.2219G>A	c.1513G>C; del(17)(q21.2)	c.1579A>G; c.196 + 1G>A
Zygosity	Biallelic	Biallelic	Heterozygous (de novo)	Heterozygous (de novo)	Heterozygous (de novo)	Heterozygous (de novo)	Heterozygous (de novo)	Heterozygous (de novo)	Heterozygous (de novo)	Heterozygous (de novo)	Heterozygous (de novo)	Heterozygous (de novo)	Heterozygous (de novo)	Heterozygous (de novo)	Heterozygous (de novo)	Heterozygous (de novo)	Biallelic	Biallelic
Sex	F (4)	M	M	M	M	F	F	F	F	F	M	M	M	F	F	M	M	M
Age of onset	8 years (range 4–12)	24 years	4 months	7 months	11 months	12 months	5 months	2 months	2 months	6 months	3 months	3 days	12 months	Stillborn	5 months	5 months	Birth	5 weeks
Initial symptom	Seizures	Seizures	Hypotonia	Development delay	Infantile spasms; Lip smacking	Seizures	N/A	Abnormal hand movements	Infantile spasms	Development delay	Seizures	Seizures	Speech delay	N/A	Development delay	Development delay	Obstructive apnoeic spells	Seizures
Epilepsy/EEG abnormalities	Yes	Yes	No	Yes	Yes	Yes	Yes	Yes	Yes	Yes	Yes	Yes	No	N/A	Yes	Yes	Yes	Yes
Intellectual disability	Yes	Yes	N/A	Yes	Yes	Yes	N/A	Yes	Yes	Yes	Yes	Yes	Yes	N/A	Yes	Yes	Yes	Yes
Slowing of developmental progress	No	No	Yes	Yes	Yes	Yes	Yes	Yes	Yes	Yes	Yes	Yes	Yes	N/A	Yes	Yes	Yes	Yes
Ataxia	Yes	Yes	No	No	No	Yes	Yes	Yes	Yes	Yes	Yes	No	No	N/A	No	No	No	No
Myoclonus	Yes	Yes	No	N/A	No	Yes	No	No	No	Yes	No	No	No	N/A	No	No	Yes	No
Brain MRI	Cerebral and cerebellar atrophy	Cerebral and cerebellar atrophy	Normal	White-matter gliosis	Microcephaly, skull and brain asymmetry	N/A	Normal	Microcephaly	Microcephaly	Asymmetric volume loss throughout the left hemisphere	Microcephaly	Cerebral and cerebellar atrophy	Microcephaly, simplified gyration	N/A	Microcephaly	Enlarged lateral and 3rd ventricles	Brain atrophy	Atrophy of brainstem and cerbellum
Dysmorphic features	Prognathism	N/A	No	No	Plagiocephaly	Prognathism	No	N/A	Micrognathia, bitemporal narrowing	No	N/A	No	Prognathism	Micrognathia, hypertelorism	No	No	Overlapping fingers	Mild
Additional features	N/A	N/A	Scoliosis, pectus carinatum, laryngomalacia	Amelogenesis imperfecta, clubfoot	Scoliosis	N/A	N/A	Severe dystonia	Optic atrophy	N/A	N/A	N/A	N/A	N/A	N/A	Autism, hyperactivity	Recurrent aspiration pneumonia, osteoporosis	Attention deficit disorder

We next pooled genetic and clinical de-identified data of patients with severe developmental disorder (DD) enrolled in the Italian Undiagnosed Rare Diseases Network (IURDN) (*n* = 110)^30^ and the Deciphering Developmental Disorders study (*n* = 13 462)^47^ and identified 3 individuals harbouring the *de novo* variants c.1652G>A (p.G551E) (Fam. IV.1) and c.2219G>A (p.R740Q) (Fam. V/VI.1) heterozygous variants in *ATP6V0A1* (Fig. 1A). Lastly, we included in the study 9 additional individuals with unresolved DD who underwent diagnostic WES through GeneDx, Peking University First Hospital and Pitié Salpêtrière University Hospitals, carrying the c.1429T>C (p.S477P) (Fam. III.1), c.2219G>A (p.R740Q) (Fam. VII-XII.1) and c.2411G>A (p.R804H) (Fam. XIII/XIV.1) *de novo ATP6V0A1* variants (Fig. 1A). We confirmed the presence of the mutations detected by WES in all individuals by Sanger sequencing and provided segregation data in immediate-relative carriers (Fig. 1B). The mutated residues in *ATP6V0A1* are evolutionarily conserved (Fig. 1C).

**Figure 2:**
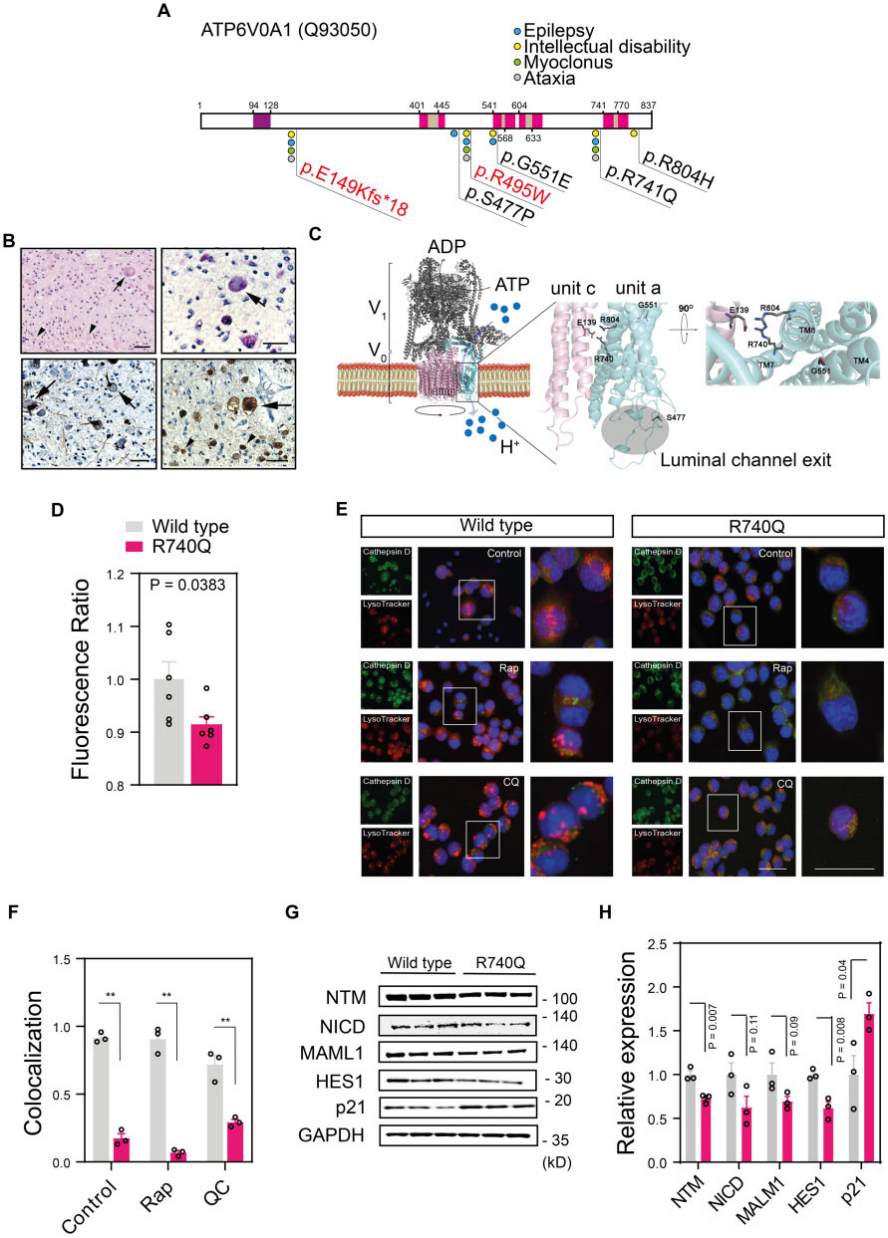
***De novo ATP6V0A1* mutations impair endolysosome acidification and activity.** (**A**) Schematic representation of human ATP6V0A1 (UniProt reference: Q93050) containing putative cytoplasmic domains (white), coil sequence (purple), transmembrane domains (magenta) and vacuolar domains (brown), according to InterPro (https://www.ebi.ac.uk/interpro/protein/UniProt/Q93050/) and localization of the identified mutations. The compound heterozygote variants are displayed in red. Amino acid numbers are indicated above and below the square boxes. (**B**) Brain histopathology of subject P: XIV.1 shows swollen neurons (arrows) and axons (arrowheads) with granular periodic acid-Schiff (PAS)-positive deposits (top left panel: haematoxylin and eosin staining; top right panel: PAS staining; bottom left panel: neurofilament light chain staining, bottom right panel: beta-amyloid precursor protein staining). (**C**) Representation of the homology model of the human V-ATPase pump and localization of the *de novo* mutations. The G551, R740 and R804 mutations in the *ATP6V0A1* gene are represented in sticks and their position on transmembrane helix (TM) 4, 7 and 8 and with respect to the protonated residue E139 of the subunit c (V-type proton ATPase 16 kDa proteolipid subunit, NP_001685.1) is magnified on the right. S477 is localized at the exit of the luminal channel. (**D**) Fluorescence intensity ratio of yellow (541 nm)/blue (441 nm) wavelengths of LysoSensor measured in Neuro2a cells expressing wild-type or R740Q mutant ATP6V0A1 mutant cells. (**E**) Neuro2a cells were treated with rapamycin (Rap 100 nM; 12 h) or chloroquine (CQ 50 µM; 12 h), preincubated with LysoTracker (red) and stained with Cathepsin D antibody (green) and DAPI (blue). Single channel (left), merged (centre), and inset (right) images are shown. Scale bars, 100 µm (merged) and 50 µm (inset). (**F**) Quantitative analysis of LysoTracker and Cathepsin D-positive compartments is shown. (**G**) Immunoblotting of R740Q Neuro2a mutant cells showed downregulation of the Notch signalling pathway, with activation of the cyclin-dependent kinase inhibitor p21. GAPDH is used as loading control. (**H**) Densitometry of the intensity of the immunoblot signals were normalized to GAPDH and expressed as fold change of R740Q mutant ATP6V0A1 samples relative to wild type. Individual data points in (**D**), (**F**) and (**H**) represent independent measurements and are displayed as mean ± SEM. *P*-values derived from unpaired two-tailed *t*-test are shown.

Altogether, we identified 17 affected subjects with mutations in *ATP6V0A1*, with p. R740Q being the most recurrent variant (8 subjects) (Figs 1A and 2A, Table 1). The mean age of onset was 11.8 ± 7.5 years for individuals carrying the compound heterozygous mutations and 5.8 ± 4.2 months for individuals with the *de novo* mutations (DNMs) (Table 1). Subjects with the *de novo* variants showed a phenotype of developmental and epileptic encephalopathy (DEE), with frequent seizures, which were refractory to treatment, and severe mental retardation after a period of apparently normal development, with delay or loss of psychomotor milestones (Fig. 2A, Table 1). Several subjects were found to have microcephaly and other facial and skeletal dysmorphisms (Table 1, Supplementary Fig. 2A and B). Brain biopsy of a stillborn infant carrying the p. R804H variant showed swollen neurons and axons, with accumulation of granular, PAS-positive material, consistent with LSD with neuroaxonal dystrophy (Figs 1A and 2B), while the placenta derived from the mother showed no evidence of storage cells (not shown).

To provide an estimate of the frequency of *ATP6V0A1* variants, we analysed the recently published predicted damaging DNMs (*n* = 45 221) from the largest cohort to date of exome sequence data from individuals with severe DD (*n* = 31 058).^31^ We observed 11 missense mutations in *ATP6V0A1*, c.2219G>A recurring in 7 patients (6 of which are included in the present study), and concluded that *ATP6V0A1* variants are the most common DNMs among all known lysosomal disease (LD) and LSD-associated genes in this database (Supplementary Table 1).

Functional studies in the well-characterized *Saccharomyces cerevisiae* V-ATPase (ScV-ATPase)^1^ have shown that the R735 residue in subunit a, corresponding to R740 in human *ATP6V0A1*, is essential for proton transport into organelles.^48^ Upon protonation of critical glutamates of subunit c, the c-ring rotates, a salt bridge with R735 is formed, and protons are transferred and pulled into the organelle lumen through a network of polar and negatively charged residues.^2^ Homology modelling suggests that the identified *de novo* mutations overall affect organelle acidification by hindering glutamate deprotonation (p.R740Q and p. R804H), deforming the architecture of the protein region devoted to protons exchange (p.G551E), or altering the conformation of the loop that contours the exit of the luminal channel (p.S477P) (Fig. 2C).

To test whether V-ATPase complex function is affected by the mutations in *ATP6V0A1*, we generated Neuro2a cell lines stably expressing wild type or c.2219G>A (p.R740Q) mutant human *ATP6V0A1* and assessed acidification of the endolysosomal compartment using the LysoSensor radiometric probe, which undergoes a pH-dependent emission shift to longer wavelengths in acidic environments. Compared to wild-type, we observed a decrease in red-shifted fluorescence signal in mutant ATP6V0A1-expressing cells, indicative of impaired protonation (Fig. 2D). Co-localization of lysosomes with Cathepsin D, an enzyme whose trafficking to the endolysosomal compartment and maturation into an active lysosomal aspartyl protease requires an acidic pH,^49–51^ was nearly completely abolished by the mutant ATP6V0A1 (Fig. 2E and F). Furthermore, western blot analyses showed decreased proteolytic maturation of Preprocathepsin into the active 31kD form upon autophagy induction with serum starvation or rapamycin treatment in mutant cells compared to wild type (Supplementary Fig. 3A and B). Intriguingly, mutations resulting in the inactivation or mislocalization of Cathepsin D lead to neuronal ceroid lipofuscinosis, suggesting a convergent pathogenic mechanism with other LSDs.^52^^,^^53^ Autophagosome turnover, a process dependent on vacuolar acidification,^54^ was also impaired in cells expressing mutant ATP6V0A1, as suggested by the increased ratios of LC3-II to LC3-I and LC3-II levels alone upon autophagy induction, which persisted after removal of rapamycin (Supplementary Fig. 4A and B). We further investigated the impairment of endolysosomal acidification in mutant cells, by assessing Notch signalling, a critical pathway for many cellular processes^55^ and dependent on V-ATPase proton pump activity for its maturation.^56–58^ We observed downregulation of Notch signalling in R740Q cells, with selective reduced expression of Notch target HES-1 and not of the transcriptional coactivator MALM1, and consequent de-repression of cell cycle inhibitor p21 (Fig. 2G and H).^59^^,^^60^

**Figure 3:**
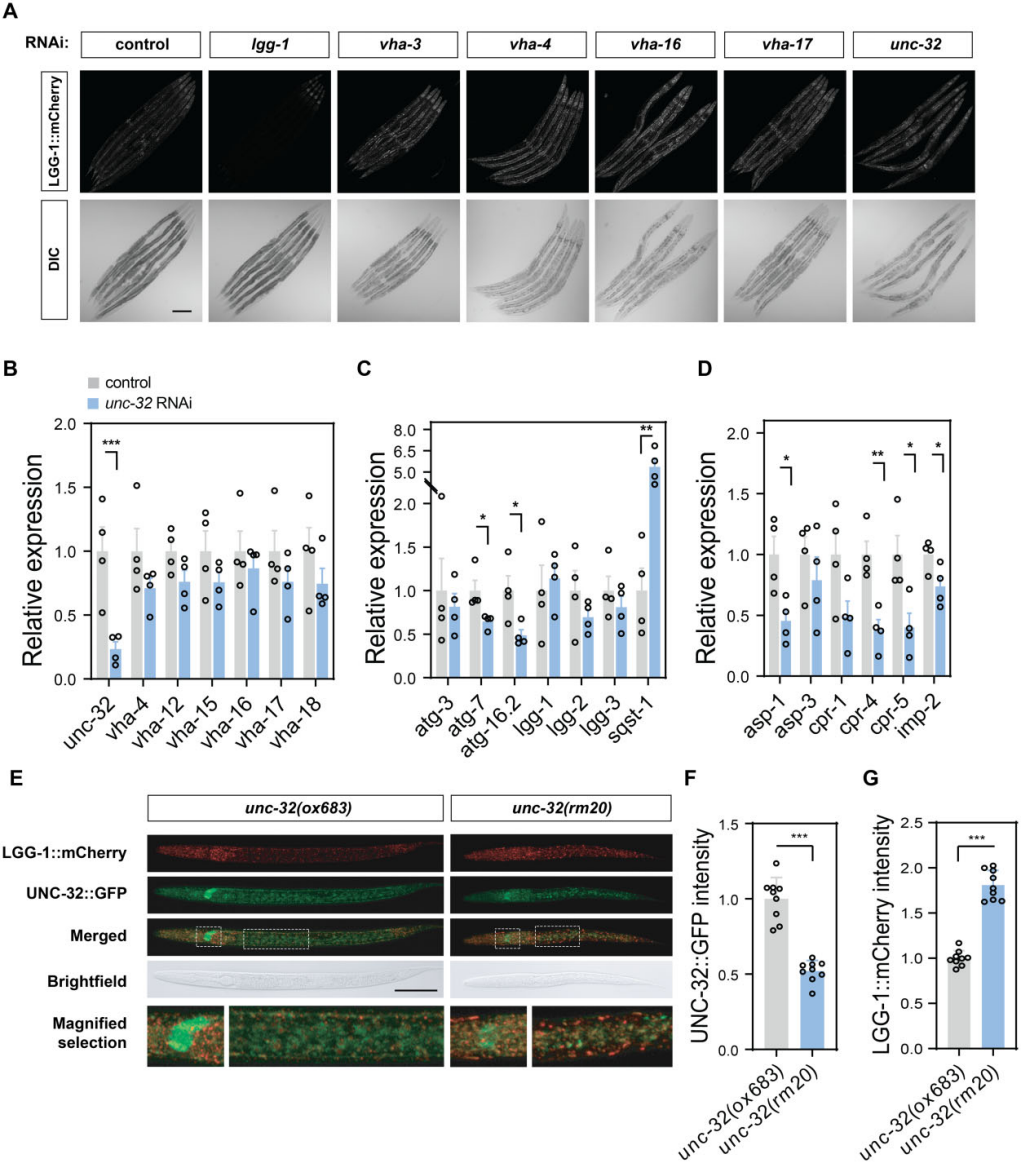
**Loss of the V-ATPase a1 subunit *unc-32* causes widespread autophagy defects in *C. elegans.*** (**A**) Confocal micrographs of age-synchronized adult animals expressing mCherry-tagged LGG-1 after treatment with RNAi targeting *lgg-1*, indicated V-ATPase genes or empty vector control. Scale bar, 200 µm. (**B–D**) Expression analysis of indicated genes relative to *cdc-42* in wild-type animals grown on *unc-32* RNAi normalized to vector control. (**B**) Expression level of V-ATPase subunit genes belonging to the V0 (*unc-32*, *vha-4, vha-16, vha-17*) and the V1 domain (*vha-12, vha-15, vha-18*) after *unc-32* RNAi treatment. (**C**) Expression level of genes belonging to the autophagic machinery after *unc-32* RNAi treatment. (**D**) Expression level of lysosomal enzymes in animals treated with *unc-32* RNAi. Individual data points represent independent experiments from 4 animals and are displayed as mean ± SEM. *P*-values derived from unpaired two-tailed *t*-test are indicated with one or more asterisks (*P < 0.05, **P < 0.01, ***P < 0.001). (**E**) Representative confocal micrographs of *unc-32(ox683) and unc-32(rm20)* larvae expressing GFP-tagged wild-type or mutant UNC-32, respectively, as well as LGG-1::mCherry. Animals were imaged 24 h post egg-lay, and the head and centre region are shown as magnified selection (dotted lines). Scale bar, 50 µm. (**F**) Quantification of UNC-32::GFP fluorescence intensity levels at the nerve ring in *unc-32(ox683)* and *unc-32(rm20)* animals. (**G**) Quantification of LGG-1::mCherry fluorescence intensity levels in *unc-32(ox683)* and *unc-32(rm20)* animals. Individual data points represent independent experiments from 9 animals and are displayed as mean ± SEM. *P*-value derived from unpaired two-tailed *t*-test is reported with asterisks (***P < 0.001).

We turned to the nematode *C.elegans* to extend our observations in an animal model of LSD.^61^ Among the genes coding for V-ATPase subunit a in *C. elegans*, *unc-32* is most closely related to human ATP6V0A1 at protein level^62^ (56% sequence identity and 69% similarity; Fig. 1C). *unc-32* is expressed throughout development and adulthood^63^^,^^64^ in multiple tissues, with highest levels in neurons.^63^^,^^64^ To evaluate the role of *unc-32* in lysosomal function, we engineered a strain to express endogenous LGG-1 (the worm ortholog of Atg8/LC3) fused to mCherry red fluorescent protein using CRISPR/Cas9. Consistent with a role of the V-ATPase complex in the autophagic pathway, RNA interference (RNAi) targeting unc-32 or other V0 subunits (*vha-3*, *vha-4*, *vha-16* and *vha-17*) increased LGG-1::mCherry fluorescence levels relative to control (Fig. 3A). Knock-down of *unc-32*, without altering the levels of other subunits of the V-ATPase complex (Fig. 3B), reduced the expression of components of the autophagic machinery (*atg-7, atg-16.2*) (Fig. 3C) and lysosomal hydrolytic enzymes (*asp-1*, *cpr-4*, *cpr-5*, *imp-2*) (Fig. 3D), and increased expression of the stress-responsive autophagy receptor *sqst-1* (Fig. 3B). Interestingly, *lgg-1* expression was unchanged by *unc-32* RNAi, indicating that the observed increase of LGG-1::mCherry levels (Fig. 3A) is caused by reduced clearance of this protein by the lysosome. We also performed Cas9-mediated editing of *unc-32* in a strain that has *gfp* inserted at the endogenous locus to introduce a point mutation corresponding to R740Q in human ATP6V0A1 (Supplementary Fig. 5A–C). Animals homozygous for the mutant *unc-32(rm20)* allele show developmental arrest at early larval stages (Fig. 3E), reduced levels of UNC-32::GFP at the nerve ring (Fig. 3F), as well as a widespread accumulation of enlarged LGG-1::mCherry puncta (Fig. 3G), further demonstrating the essential role of V-ATPase activity in organismal health.

## Discussion

Here, we report 17 subjects from 14 unrelated families carrying *de novo* missense or inherited biallelic variants in the *ATP6V0A1* gene, representing the largest cohort to date. From a series of 115 individuals with Rett or Rett-like syndrome, a *de novo* p. R741Q change in *ATP6V0A1* (corresponding to p. R740Q according to the updated reference sequence NM_001130021.3 used in this manuscript) was first identified by trio-based WES as a variant of uncertain significance in one proband with gait abnormalities, stereotypic hand movements and partial loss of hand skills.^65^ Aoto et al.^66^ recently reported 4 individuals, two with the same p. R741Q *de novo* variant (corresponding to NM_001130021.3: p. R740Q) and two with the following biallelic mutations: a p. A512P missense variant (corresponding to NM_001130021.3: p. A505P) in compound heterozygosity with a 50-kb deletion, and a p. N534D missense variant (corresponding to NM_001130021.3: p. N527D) in compound heterozygosity with a disruption of a splice donor site. All the *de novo* cases reported so far displayed a phenotype of developmental and epileptic encephalopathy (DEE), with developmental delay in the first months of life and drug-resistant focal and generalized tonic-clonic seizures. Head circumference 2 standard deviation below the average was present in 50% of the children with the p. R740Q variant and one individual with the p. R804H variant, suggesting an association between microcephaly and *ATP6V0A1*-related diseases. With 58 DNMs (50 missense, 3 indel, 2 splice site, 2 initiator codon and 1 frameshift) in genes of the V-ATPase complex identified in exome sequence data from over 30 000 individuals with severe DD,^31^ and significant association with severe DD for 2 genes of this group (*ATP6V0A1* and *ATP6V1A*) out of 281, we propose that a V-ATPase-related disease should be considered in individuals presenting with mild-to-profound developmental delays and epilepsy, with or without microcephaly. Five individuals from 2 unrelated pedigrees in our cohort carry the biallelic E149Kfs*18 frameshift and R495W substitution and manifest an early onset, progressive myoclonus epilepsy (PME) and ataxia, therefore, expanding the neurological phenotypes associated with *ATP6V0A1* variants beyond the more severe developmental and epileptic encephalopathy observed in the *de novo* cases. Myoclonic epilepsy was also a feature in one of the biallelic cases recently reported.^66^ Notably, the recessive cases were all characterized by cerebellar atrophy in the brain MRI, clinically resulting in mild to prominent ataxia, suggesting that the cerebellum is more commonly affected in the recessive cases compared to the *de novo*.

The *ATP6V0A1* gene encodes the a1 isoform of the membrane-bound V0 components of the V-ATPAse complex, which of the four a-subunits is the most neuronal enriched,^67^ accounting for the predominantly neurological phenotype observed in these patients. In contrast with other subunits of the membrane V0 components, the a isoforms are encoded by different genes and display only approximately 50% sequence homology.^68^ Recent cryo-electron microscopy studies combined with mass spectrometry analysis of human V-ATPase, showed that the membrane-embedded C-terminal domain of subunit a1 form the surface in contact with the ring of c subunits (c-ring) and creates the two offset half-channels that allow the passage of the protons.^2^ Rotation of the c-ring brings a protonated glutamic acid close to R740 of subunit a1, with formation of a salt bridge between the residues and release of the proton into the luminal half channel.

The *de novo* variants identified here cluster on the transmembrane domains of the a1 subunit and are predicted by homology modelling studies to hinder glutamate deprotonation (p.R740Q and p. R804H), deform the architecture of the protein region devoted to protons exchange (p.G551E), or alter the conformation of the luminal channel (p.S477P), suggesting that perturbation of proton translocation is fundamental to the disease (Fig. 2A and B). Intriguingly, the R495W substitution from the biallelic series results in loss of a positively charged hydrophobic residue in close proximity of S477 and the luminal channel exit. We speculate that this variant has a less damaging impact on the proton pumping activity, leading to neurological manifestations only when in compound heterozygosity with a null allele. Of note, the parents carrying only one of the variants do not manifest any obvious neurological phenotype, suggesting a mechanism of haploinsufficiency for the inherited variants.

We next proceeded to study the mechanism of pathogenicity of the R740Q substitution, which accounts for over 70% of all *de novo* cases reported so far, effectively representing a mutation hotspot. Our experimental work indicates that the R740Q variant causes altered organelle acidification and failure of lysosomal hydrolysis directly by impairment of the canonical proton-pumping V-ATPase function. The compromised acidification of the endolysosomal compartments leads to impaired γ-secretase-mediated processing and release of the Notch intracellular domain (NICD) and reduced expression of Notch target genes. These findings corroborate previous reports in *Drosophila* indicating that the V-ATPase-mediated acidification of the endolysosomal compartment is required for the activation of Notch in endosomes^69^ and expression of a dominant negative subunit of V-ATPase in neural precursors reduced Notch signalling and depleted the neural stem cell niche.^70^ Overexpression in neuronal cells of ATP6V0A1 constructs carrying the A512P (NM_001130021.3: p. A505P) and N534D (NM_001130021.3: p. N527D) variants also resulted in impaired lysosomal acidification, as recently shown,^66^ suggesting that impaired protonation is a shared mechanism among other disease-associated variants. Confirming these observations in disease models where the mutant and wild type allele are expressed at physiological levels is required in future studies to conclusively establish a critical pathogenic role of the disruption in endolysosome acidification in these diseases.

To further establish a role of this isoform in lysosomal function, we knocked down the *ATP6V0A1* ortholog in *C. elegans* and observed reduced protein clearance in lysosomes and altered regulation of several components of the autophagic machinery in neurons, expression of global autophagy failure. Homozygote nematodes carrying the corresponding R740Q mutation showed severe developmental arrest at early larval stage, with widespread accumulation of undigested proteins in the nerve ring. In accordance with recent findings of lysosomal dysfunction and increased neuronal cell death observed in brains of ATP6V0A1 mutant mice carrying the homozygote A512P change,^66^ overall these results show that ATP6V0A1 is critical for brain development and support an antimorphic mode of action of the R740Q and the other *de novo* variants.

In conclusion, here we identified *de novo* and biallelic variants in *ATP6V0A1* in patients affected by DEE and PME and link the mechanism of pathogenesis to disruption of the canonical protonation function of the V-ATPase complex. Considering the increasing identification of disease-causing variants in V-ATPase genes and the critical relevance of vacuolar pump-mediated regulation of intracellular pH in cellular homeostasis in health and disease,^4^ this multi-protein complex is rapidly taking centre stage as a molecular hub for unravelling the disease mechanisms of LDs and other human diseases, from cancer to neurodegeneration.

## Supplementary material

Supplementary material is available at *Brain Communications* online.

## Supplementary Material

fcab245_Supplementary_Data

## Appendix

**Italian Undiagnosed Diseases Network:**

Domenica Taruscio, Marco Salvatore, Agata Polizzi, Federica Censi, Giovanna Floridia, Giuseppe Novelli, Erica Daina, Alessandra Ferlini, Marcella Neri, Dario Roccatello, Simone Baldovino, Elisa Menegatti.

**V-ATPase Consortium:**

Nancy Pinnell, Dallas Reed, Peter D Turnpenny, Jacqueline Eason, Leah Fleming, Kirsty McWalter, Kali Juliette, Paul J Benke, Xilma Ortiz-Gonzalez, Sarah Mckeown, Amisha B Patel, Matthew Osmond, Jagdeep S Walia, Xianru Jiao, Zhixian Yang, Boris Keren, Charles Perrine, Ashish Deshwar.

## Abbreviations

DD: developmental disorder
DEE: developmental and epileptic encephalopathy
DNM: *de novo* mutation
HES1: hairy and enhancer of split 1
LD: lysosomal disease
LSD: lysosomal storage disease
MAML1: mastermind-like 1
NICD: otch intracellular domain
NTM: notch transmembrane
PME: progressive myoclonus epilepsy
V-ATPase: vacuolar-type ATPase
